# When normality collapses from one moment to the next. A sociological theory of singular crisis

**DOI:** 10.3389/fsoc.2025.1596427

**Published:** 2025-07-04

**Authors:** Klaus Kraemer, Joris Steg

**Affiliations:** ^1^Department of Sociology, University of Graz, Graz, Austria; ^2^Department of Sociology, University of Wuppertal, Wuppertal, Germany

**Keywords:** crisis, singular crisis, normal crisis, normality, COVID 19, sociology, social change

## Abstract

Since the emergence of sociology, it has been part of the discipline’s self-image to diagnose crises in modern societies. Sociology, however, has no theory that differentiates between normal and extranormal or singular crises. In this article, we want to develop a crisis typology that distinguishes between these two types. While a normal crisis is characterised by cyclical and structural patterns, which usually build up gradually and lead to incremental change, a singular crisis is characterised by eruptive ruptures in relation to the pre-crisis state. Such ruptures can challenge the traditional social order, both institutionally and narratively. Unlike normal crises, a singular crisis is marked by exogenous shocks like wars, natural disasters, or pandemics. This shock marks the beginning of a process of crisis intervention, which we examine to reconstruct the sociological peculiarities of a singular crisis. By using the Covid-19-crisis as an empirical slide, we analyse a singular crisis and list various dimensions and criteria—namely involvement and impact, temporality, principle of order, social change, isomorphism, path dependency, collective morality, mode of legitimation and spatial order—that can be used to differentiate between singular and normal crises.

## 1 Introduction

We are living in times of crisis. The term crisis is currently omnipresent, it is used almost inflationary. There is probably no other term that characterises and dominates the topical media, political, scientific and public discourse in a comparable way. The fact that we are living in a crisis-ridden and critical time of upheaval and change is now a commonplace. And indeed, a cursory glance at various crisis phenomena of the last two decades illustrates the ubiquity of crises in European and American societies: The global banking, financial and economic crisis of 2007–2009, which seamlessly transitioned into the sovereign debt and euro crisis from 2010, was followed by the so-called refugee and migration crisis in 2015. With the election of Donald Trump as US President and the Brexit in 2016, a crisis of liberal democracy, characterised by the global rise of nationalism, right-wing populism and authoritarianism, was then widely noted. At the latest with the emergence of the Fridays for Future movement, the climate change moved into the collective consciousness. Finally, in March 2020, the coronavirus crisis broke out, in which fundamental basic rights were suspended and restrictions on public and private life that had been deemed impossible were imposed by state authorities.

Covid-19, in turn, was by no means the end of the cascade of crises, as Russia’s invasion of Ukraine in February 2022 brought the war back to Europe. The cascade of crises and the war resulted in rapidly rising inflation rates and a veritable energy (price) crisis in Europe. In October 2023, the Middle East conflict escalated once again: following the terrorist attack on Israel by the radical Islamic organisation Hamas, the Israeli army launched a massive military operation in the Gaza Strip, triggering another war in the Middle East. In view of increasing refugee and migration movements since 2022, there has also been renewed talk of a “migration crisis.” In recent years, extreme weather phenomena such as droughts, water shortages and heatwaves on the one hand and heavy rainfall and flooding on the other have served as a tangible reminder of the urgency of climate policy measures.

Modern societies seem to be confronted with increased and accelerating crises. Various crises that occur simultaneously, overlap, are mutually dependent and reinforce each other in their effects. While some call this constellation a “polycrisis” (Juncker, 2016; Tooze, 2021; Lawrence et al., 2022; Lawrence et al., 2024), a “perma-crisis” (Brown et al., 2023) or a “mega-crisis” (Boin et al., 2021a) to characterise the present, which is shaken by an exceptional cascade of crises, others speak of the “new normal” (Ashton and Toland, 2021) to describe the crisis-induced changes. Even if the current crisis configuration is conceptualised differently: What is certain is that the crises and the crisis-like nature of modern societies are at the centre of political, media, public and academic attention. Crisis seems to have developed into “a structural signature of modernity” (Koselleck, 2006, p. 372). Crises are constantly being diagnosed everywhere. “Crisis is an omnipresent sign in almost all forms of narrative today” (Roitman, 2014, p. 3). Crises are phenomena that characterise the mentality and structure of current societies. We live in a time characterised by disorder, insecurity, uncertainty, upheaval, transformation and contingency.

Sociology as a science of society is always called upon and challenged, when things deviate from the norm and anomalies can be observed, when social upheavals and changes occur, when social dysfunctions and pathologies threaten, and especially when critical developments intensify and crises escalate. In times like these, sociology should offer interpretations, provide orientational knowledge and be able to discuss the specific characteristics of current dynamic crises as well as similarities and differences to previous crises in a theoretical manner and examine them empirically. Since the emergence of sociology, it has been part of the discipline’s self-image to diagnose crises in modern societies. It is noteworthy, however, that sociology does not have an adequate theory of social orders that distinguishes ‘normal’, i.e., recurring crises from extranormal or singular crises.

In this article, we want to develop a crisis typology that distinguishes between singular or extranormal crises and normal crises. On the one hand, a theory of singular crises expands the conceptual and analytical tools of sociology and enables the empirical analysis of various crises. On the other hand, we believe that a theory of singular crises can also contribute to a better understanding, explanation and sociological classification of social and institutional change under different crisis conditions. Because crises are sometimes highly dynamic, trigger far-reaching cascading effects under unique conditions and can even temporarily promote social differentiation, a process-based understanding of crises is necessary.

We will proceed as follows: in the second section we will shed light on the emergence of sociology as a science of crisis. Then, in the third section, we identify core definitional features for a sociologically appropriate understanding of crises. In the fourth section, we look at normal crises and explain the need to differentiate between different types of crises. In the fifth section, we use the example of the Covid-19-crisis as an empirical slide to analyse a singular crisis. We analyse what makes the corona crisis so unique and singular and we list various dimensions and criteria that can be used to differentiate between singular or extranormal crises and normal crises. In doing so, we draw on selected classical and more recent theoretical approaches and authors in sociology who we believe could contribute to a better understanding of these singular crises, which have largely been overlooked in the social sciences thus far. Finally, in the sixth section, we draw some theoretical conclusions on a sociology of singular crises and problematise the extent to which a singular crisis can trigger social and institutional change.

## 2 The emergence of sociology as a science of crisis

Although the term crisis is used in an almost inflationary manner, it is not easy to define.^1^ The history of the term crisis dates back to ancient Greece. The etymological origin of crisis lies in the Greek noun krísis, which can be translated as dispute, judgement, assessment, distinction, decision, turning point or also culmination, and the complementary verb krínein, which can be translated as to examine, separate, (sub)divide, decide, (be)judge, argue or fight and also forms the etymological origin of the term critique. In antiquity, the term krisis was used in various contexts and areas of society to describe different phenomena. In politics, krisis referred to decisions, resolutions and political disputes; in a legal-forensic perspective, krisis referred to court proceedings and, in particular, to the passing of judgement. This meaning was also reflected in the theological context, where the term referred to the judgement of God and the Last Judgement. However, the primary field of application of the term crisis in ancient Greece was the medical field. Here, crisis referred to the final phase of the disease process, in which it was decided whether recovery would occur or not, i.e., the phase in which the decision on death or survival was made.

Even though there have been crises in all phases of social development, crisis as an instrument of social self-description and as a scientific category of analysis is a phenomenon of modern societies. Since the 19th century, it was the periodically recurring economic crises in the emerging industrial capitalism as well as the epochal social modernisation, upheaval and transformation processes that led to the metaphorical expansion of the semantics of crisis, contributed to the growing importance of the term of crisis for the description of social conditions and accelerated the further scientific examination of the concept of crisis.

It is therefore not surprising that it was precisely at this time that sociology emerged as an independent academic discipline. The central impulse for the genesis of sociology was among other the observation of multiple crisis phenomena and the serious social upheavals in the 19th century. Sociology has historically constituted and established itself as a science of crisis (Repplinger, 1999; Steinmetz, 2017; Steg, 2020, 2023). Sociology emerged explicitly as a science that should deal with the fundamental crisis nature and the specific crises of modern societies.

According to the widespread self-understanding of the discipline, crises in social orders, regardless of their form or magnitude, are investigated. Since the sociological classics, there has been broad consensus that modern societies can be described as fragile orders in which phases of relative stability are repeatedly replaced by crises. From this perspective, crises are not interpreted as historical accidents or anomalies, but as ordinary and normal recurring conditions of modern times. The self-understanding of the discipline as a science of crisis was constituted by the diagnoses of crisis made by the sociological classics Karl Marx, Émile Durkheim, Max Weber and Georg Simmel, who examined the crises and crisis-proneness of emerging modern societies from different perspectives and with different theoretical approaches.

In his analysis of the anatomy of bourgeois society, Marx (1990, 1992a, 1992b) focussed on economic crises, which he saw as an inherent and cyclically recurring feature of the capitalist mode of production, and described the social consequences of this socio-economic crisis diagnosis as processes of exploitation and alienation of the working class. In France, Durkheim (1984, 2002) investigated the social problems of modern industrial society and thus founded the influential sociological theory of anomie. He primarily emphasised the cultural-normative dimension in his diagnosis of crisis and concentrated on disruptions to social cohesion, loss of community, social pathologies and anomies. Simmel (2004) problematised the social paradoxes of the modern money-driven economy and interpreted these as a widening gap between “objective” and “subjective culture.” In a very similar way, Weber’s (1978) epochal thesis of “occidental rationalization” diagnosed a differentiation of value spheres. Weber’s diagnosis of the crisis, however, had a primarily institutional-bureaucratic-political character. According to Weber, the systematic processes of bureaucratisation and rationalisation in the state, capitalist economy and society lead to a loss of meaning and freedom as well as a loss of individuality and personality. Weber thus shaped the way generations of sociologists in the 20th century described the permanent crisis of modern societies like no other. Since the analyses of the sociological classics, the diagnosis of a crisis of modern society has been a continuity in sociology.

The tradition of sociological analysis of the vulnerability of modern societies was continued in the course of the 20th century by Robert Merton and Pierre Bourdieu. Merton (1938, 1968) developed the model of “relative deprivation” based on Durkheim’s theory of anomie to explain the social problems of North American society from the 1930s to the 1960s in terms of the discrepancy between collectively shared cultural goals such as “success,” “achievement” and “prosperity” and the insufficient means available to individuals to legitimately achieve these goals. Bourdieu (1984) ultimately built on this model and translated it into a social theory of habitus, which he used to explain the growing social tensions in the French education system of the 1960s and 1970s (“hysteresis effect”). And finally, reference is made to the long and influential tradition of social science research into the structurally and cyclically recurring crises of modern capitalism, which have been investigated in the tension between destruction and innovation (Schumpeter, 1942), disembedding and embedding (Polanyi, 1944), prosperity and legitimation crises (Habermas, 1992), democratisation and de-democratisation (Streeck, 2014; Brown, 2015), or equality and inequality (Piketty, 2014, 2020; Milanovic, 2016).

Even in recent times, a large number of authors have been competing for the sociological prerogative of understanding and explaining the critical upheavals of the early 21st century. Exemplary are the analyses of the crisis of democratic capitalism (Streeck, 2011, 2014; Wolf, 2023) and the crisis of liberal democracy (Levitsky and Ziblatt, 2018; Merkel and Kneip, 2018; Mounk, 2018; Runciman, 2018; Przeworski, 2019; Calhoun et al., 2022). Exemplary are also analyses of new value conflicts around culture and identity (critically cf. Kraemer, 2020), the digitalisation of the social in the “ordinal society” (Fourcade and Healy, 2024), to social divide and social polarisation (Hetherington and Weiler, 2018), or to the challenges of the climate change and a socio-ecological transformation (Beckert, 2025).

## 3 What does crisis mean?

Although crisis plays a central role in the (history of) sociology, no uniform understanding of crisis has yet emerged. The concept of crisis is also not easy to define. The mere fact that the concept of crisis can refer to practically all social and individual problems makes it difficult to find a universally valid, binding and undisputed definition of crises. The fundamental problem and central difficulty in defining the concept of crisis lies in determining when exactly a crisis exists, when exactly an event or a development is justified to be categorised as a crisis. In any attempt to define the concept of crisis, it must be noted that there is no standardised and undisputed definition of the concept of crisis that is valid for all conceivable crisis phenomena and specifies critical threshold values for the transition from normal to crisis-like conditions. Nevertheless, some core definitional features of crises and central building blocks for a sociologically appropriate understanding of crises can be identified (Steg, 2020, 2023; see also Kiess et al., 2023).

In general, crises can be described in a minimal definition as escalating decision-making phases with a basically open outcome. Crises always refer to the temporary deviation from normality and the desired state; they denote the unintended deviation from the normal, problem-free functioning and development of a social organism or a social system. In this respect, crises are always a relational category that refers to the negation or aberration of a reference identified as normal. Even if a crisis can last a long time, it can never be a permanent or normal state. Crises are by definition finite and temporary. Every “crisis concerns a temporal period that is short relative to those that precede and follow” (Walby, 2015, p. 20). If crisis was the normal or the permanent state, the concept of crisis would no longer be necessary, as crisis would be identical to normality. If everything is a crisis and if there is always a crisis, then nothing is a crisis. Crises are, especially in modern capitalist societies, indeed normal and common social phenomena, insofar they are irrevocable components of social development, but nevertheless each crisis in itself represents a non-normal, non-planned, non-desired and temporally limited exceptional situation.

Crises are always the result of preceding processes and the preliminary stage of future processes; crises are thus both a product of development and a producer of development. Crises represent transitional phases that point to a contingent future. Because their consequences are not predetermined and their outcome is open, crises systematically produce a moment of ambiguity, uncertainty, and insecurity. However, crises are not only phases of uncertainty and insecurity, but also of unsecuring and repeal. Crises can discredit and delegitimise old, self-evident truths and traditional beliefs. As a result, crises create a veritable pressure to act and adapt. Crises are therefore decision-making situations in two ways: On the one hand, in acute crisis phases, decisions must be made under time pressure to deal with the crisis; on the other hand, crises represent crossroads and junctures in which the further course of development of the phenomenon in crisis is decided.

Crises enable alternative paths of development that would not be possible without the “window of opportunity” that a crisis opens up. In a crisis, “there is the possibility of large-scale change” (Walby, 2015, p. 34). Social change is hardly conceivable without crises. Crises can follow social change. They can also precede social change. Crises can trigger incremental change over time, as shown by international comparative research on varieties of capitalism (Hall and Soskice, 2001) or research on welfare worlds (Esping-Andersen, 1990). Or they can accompany, accelerate, mitigate, delay or even block social change. The most diverse forms of the interrelationship between change and crisis are theoretically possible and can also be empirically plausible. Therefore, over-simplistic cause-and-effect explanations are out of the question.

Even if the outcome of crises is open and contingent, the concept of crisis should not be trivialised with an everyday linguistic and scientific arbitrariness. Crises can be seen as “a serious threat to the basic structures or the fundamental values and norms of a system, which under time pressure and highly uncertain circumstances necessitates making vital decisions” (Rosenthal et al., as cited in Boin et al., 2018, p. 24). However, one can only speak meaningfully of a crisis if a situation is also perceived as crisis-like by the members of society. The diagnosis of a crisis and the reception and perception of a crisis must coincide at least to a certain extent. In other words, a crisis diagnosis must find its way into the public sphere—via experts, the media or other actors—and there must be a crisis narrative. The crisis narrative, in turn, must have empirical content, the material character of the crisis must be given. And finally, there must be an audience. An audience that believes and takes up the crisis narrative of the crisis spokespeople. “Thus, only when members of a society experience structural alterations as critical for continued existence and feel their social identity threatened can we speak of crises” (Habermas, 1992, p. 3). And this unity of speaker and audience must extend far into the space of public representation. The famous Thomas theorem also applies here (Thomas and Thomas Swaine, 1928). Without actors appearing and defining a social reality as crisis-like and without such a definition of the situation being collectively believed by many others, there can be no crisis. If nobody talks about a crisis and nobody perceives a constellation as a crisis, then it does not exist.

To summarise, we define a crisis as a non-intended deviation from normality. Not every deviation or process of change is a crisis, crises are situations in which there is a critical, potentially existential threat to the structure, functionality or legitimacy of a social system, a social organism or a social context. Crises always create pressure to act and adapt. Under conditions of uncertainty and time pressure, decisions must be made to overcome the crisis. In addition, a number of conditions must also be met to speak meaningfully of a crisis: First of all, a situation must be defined as a crisis and a crisis narrative must be conveyed. Then, this crisis diagnosis must be collectively believed and shared. There must therefore be a unity of diagnosis, reception and perception of a crisis.

## 4 Normal crises

Sociology has a large repertoire of crisis diagnoses and theories. Despite their differences, what they all have in common is that they focus on “normal” crises. Because crises in modern capitalist societies have their root causes in systemic-structural conditions, they are integral components of social development. From a sociological perspective, such crises are “normal” even if they are perceived by contemporary actors as extraordinary. As frequent social phenomena, “normal crises” come and go. They build up imperceptibly, as in the example of over-and subordination (Simmel, 2009, p. 129–226), injustice (Moore, 1978), creeping economic crises (Boin et al., 2021b), the “Minsky moment” (Minsky, 1982) on financial markets or gradually intensifying poverty crises (Paugam, 2005). Or they are postponed (Krippner, 2011) and “deferred” (Streeck, 2014). Such crises are obviously part of the normal development of modern societies, especially of capitalism and its “creative destruction” (Schumpeter, 1942). The normality of this type of crisis is that it flares up cyclically or structurally. In normal times, the patterns of crises are often imperceptible, insidious, and gradual.

In modern “functionally differentiated capitalist societies” (Schimank, 2015) such crises are unintended consequences of economic, political and social order and mode of development. As already pointed out, we can only speak of a crisis if it is perceived collectively. A crisis must therefore cross a specific threshold of perception. In the sociological sense, it is at best a latent crisis if there is no explicit awareness that the established institutional order is endangered or under threat (Habermas, 1992). A crisis can only be addressed in a political and institutional way and possibly trigger social change. However, crises can also slow down or even block social change. In this case, ambitious reform efforts come to nothing. The reasons for social stagnation or “immobilism” (Lepsius, 2009) may be manifold. In sociology, such phenomena of non-change are often described in theoretical terms as social persistence and are also well studied empirically.

We have shown that sociology offers a multitude of diagnoses and theories for analysing crises in modern societies. Somehow, all these crises are “normal“. But not every crisis is like the other. Even if crises share structural characteristics, every crisis is fundamentally specific. Every crisis has its own specific trigger, its own concrete course and its own particular consequences. However, crises differ not only in the specific empirical case. Different forms of crises can and must also be distinguished from one another analytically and typologically.

## 5 Singular/extranormal crises: criteria and dimensions

In terms of intensity, duration, depth, scope and their structural logic, a specific type of crisis can be distinguished from the normal crises, which we call singular or extranormal crises (Kraemer, 2022, 2023). Unlike normal crises, which unfold gradually and often allow for the maintenance of core institutional structures, a singular crisis is marked by “exogenous shocks” (Fligstein and McAdam, 2012, 99) like wars, natural disasters, or pandemics. The event of an exogenous shock marks the beginning of a social process of crisis intervention, which we will examine in more detail below to reconstruct the sociological peculiarities of a singular crisis as compared to the type of normal crisis. For our argument, it is crucial that an exogenous shock can literally turn the established social order upside down from one day to the next. We speak of a disruptive process because established practices (action) and justifications (talk) (Brunsson, 1989) are called into question literally overnight, as are institutions, social certainties, habits and epistemic beliefs that were previously valid, which in normal times provide a basic sense of security (Popitz, 2017). Singular crises are characterised by their “singular extraordinariness and radical uncertainty” (Kraemer, 2022, p. 7). These crises are times in which normality is not just temporarily interrupted but collapses from one moment to the next.

It is remarkable that such singular, extraordinary crises have hardly been systematically studied in sociology to date (see Simmel, 2009, p. 280–283; Coser, 1956, p. 87–95; Lederer, 2014, using the example of the July crisis of 1914; Münch, 2022, p. 51–61). Furthermore, the research of historical sociology (McAdam et al., 2001; Delanty and Isin, 2003) on political revolutions and the sociology of disasters (Matthewman, 2015; Tierney, 2018; Drabek, 2019) on local or regional environmental crises have not yet been systematically reviewed to develop a general sociology of singular crises. In sociology, social phenomena are usually examined along the dimension situation, action and structures. James S. Coleman (1990) “bathtub” is particularly influential for understanding the process-related nature of social phenomena and their mutual micro–macro interconnection. However, if you want to study social phenomena in extranormal times, then it is essential to consider another dimension: the dimension of an “event” (Wagner-Pacifici, 2017, see also Sewell, 1996, 2008). One could argue that singular events are unique and incomparable. In contrast to sociological approaches that do not consider historical events more systematically as an independent, irreducible dimension for the theoretical explanation of social orders, we argue that singular crises remain misunderstood if they are not examined sociologically. At the same time, we suspect that the analysis of singular crises can also help to better understand social orders in normal times.

Historians often underscore the uniqueness and incomparability of events, which can lead some to argue that such singular occurrences are not the domain of sociology. We contend otherwise. Our counter-thesis is twofold: Extranormal or singular crises, regardless of their situational singularity and the incomparability of their specific triggers and peculiarities, exhibit some distinct similarities, or more precisely structural similarities and similar social patterns. Firstly, singular crises are indeed unique, but secondly, they exhibit important similarities and recurring social patterns and social structures that render them profoundly relevant to sociological analyses. This paradox points to the importance of examining singular crises through a sociological lens. Singular crises are therefore not sociologically relevant because of the singularity of an “exogenous shock,” but because similar social patterns of collective crisis response and crisis management can be studied.

What makes a singular crisis, which is historically incomparable, sociologically comparable? The very first sociological similarity of a singular crisis is that the social reality that we take for granted in the pre-crisis, in normal times, is abruptly interrupted or even suspended in the moment of a singular crisis. From one day to the next, literally overnight, established political-institutional rules, economic practices, collectively shared interpretations of the social world, epistemic beliefs, but also the social organisation of space and time, that ordinarily appear stable and immutable, become fundamentally problematic in the face of war, natural disaster, or pandemic. In the following, we will explain in more detail the thesis on the sociology of a singular crisis using the example of the SARS-Cov2 pandemic.

Historical knowledge about previous epidemics and pandemics is certainly available (McNeill, 1976; Spinney, 2017; Honigsbaum, 2020). The methodological instruments of sociology cannot answer the question of whether the coronavirus crisis was a unique health crisis in a medical, epidemiological and infectiological sense or whether it was more of a common, periodically recurring pandemic. The singularity thesis is meant purely sociologically in the following. Our considerations merely relate to the collective social patterns of crisis response in the various fields of action or subsystems within society. In line with the classic Thomas theorem (Thomas and Thomas Swaine, 1928), the sociological classification of a crisis as normal or singular is not only based on objective data about the—presumed or actual—quantitative extent of the damage of the crisis and the danger or threat to the society, but on the way in which the crisis is collectively perceived as an existential uncertainty (Habermas, 1992) and the institutional patterns of crisis response in politics, law, economics, culture and society that build on this.

We will identify different social patterns (see also Kraemer, 2022) that make a historically unique and therefore actually incomparable crisis like the pandemic of 2020–2022 sociologically comparable. Such an approach corresponds to the basic methodological understanding of sociology. After all, sociology has always been concerned not only with describing social structures, social action and social issues, but also with categorising, understanding and explaining them and developing sociological ideal types (Weber, 1978). Thus, our aim is to formulate generalizable, ideal-typical propositions that go beyond the singular case, and to draw theoretical conclusions from them. The following remarks are not intended to identify recurring social patterns in normal times, as is usually the case in sociology. Rather, we intend to identify generalizable social patterns based on a crisis that is unique in every respect. We expect this methodological approach to provide not only a more precise sociological understanding of the unprecedented corona crisis, but also, and above all, new theoretical insights into a general sociology of singular crises. Fundamental to the following considerations on the sociological characteristics of a singular crisis is the theoretical assumption that a central feature of modern societies that is typical in normal times, namely functional differentiation, is partially suspended in terms of time, space, material and social dimensions.

### 5.1 Degree of involvement and impact

Crises have a different quality in terms of their scope, duration, intensity, dynamics, involvement and impact. While some crises remain manageable in their impact and only affect individual areas or subsystems of society, singular crises are characterised by the fact that they affect society as a whole and have a transnational and global scope. As the Covid-19 pandemic shows particularly clearly, singular crises can affect anyone, anywhere. Singular crises are defined by their highly dynamic nature and their social as well as spatial and temporal delimitation and dissolution of boundaries. Furthermore, in empirical reality, singular crises are multiple crisis phenomena. In the case of extranormal crises, we are dealing with simultaneous, overlapping, accumulating, interwoven and intensifying crisis processes. Singular crises have “cascading” effects. In addition to the triggering events, “secondary effects with a greater impact than the primary event” (Alexander and Pescaroli, 2019, p. 1) can be observed. That means that crisis phenomena in one place have an impact on crises elsewhere. Crisis management can also lead to other crises. During the Covid-19 pandemic, the measures taken to contain the pandemic led to a slump in the global economy, which in turn led to company insolvencies and closures. In addition, the school closures have had and continue to have serious psychosocial and educational consequences (Ludwig-Walz et al., 2023; Mazrekaj and De Witte, 2024).

### 5.2 Temporality

Crises have their own temporal structure and logic. But not every crisis has the same temporal structure. The temporal structure of a singular crisis differs fundamentally from the temporality of normal crises. While the signs of a crisis usually appear and accumulate in normal crises, singular crises erupt abruptly, suddenly, unexpectedly and with full force. In singular crises, it therefore is difficult to speak of a pre-crisis at all, since latent crisis symptoms can hardly be anticipated, even on the eve of the crisis escalation. In contrast to normal crises, the signs of a singular crisis do not accumulate. With the outbreak of the crisis—or rather, with the official proclamation that it is an unprecedented, singular crisis—the times change, but not gradually, but disruptively (Suckert, 2021). Singular Crises are characterised not only by their radical uncertainty and highly dynamic, but also by the fact that that there is a radical interruption or suspension of normality.

Within a very short period of time, a vague fictional scenario becomes simple reality. What was unthinkable yesterday can no longer be ignored today and will become the unquestioned “new normal” tomorrow. With the first lockdown in Europe in March 2020, the “old normal” was replaced by the “new normal” overnight. Across all social fields and subsystems, the previously valid, generally accepted normality was being replaced by a “new normality.” This applies not only to everyday normative expectations, ways of thinking and practices at the micro level, but also to political practices, organisational and institutional arrangements and value systems at the meso and macro levels. Under normal circumstances, the passage of time flows in a relatively predictable manner, enabling habitual social practices to continue largely undisturbed. However, in a singular crisis, the continuity of time is effectively interrupted—sometimes overnight. In the shock moment of the crisis, the things we take for granted and the habits of the “old normal” are suspended until further notice. In the “whiteout moment” (Kraemer, 2022) of a singular crisis, time literally seems to stand still.

The temporal structure of a singular crisis also changes the way the past and the future are perceived. All established expectations are undermined because the future becomes fundamentally unforeseeable. In normal times, latent future uncertainties are everywhere. In a singular crisis, however, future uncertainties are no longer just latent, but overwhelming. They escalate into the boundless. One finds oneself in an “expectation vacuum” (Kraemer, 2021). The temporal horizon of expectations shrinks to surviving the crisis test in the here and now. Under these conditions, neither past nor future-oriented expectations provide social orientation. In normal times, past-oriented expectations (norms, conventions, classifications) have proven themselves many times over. In a singular crisis, such expectations scarcely generate certainty for action or ontological security. The same also applies to future-oriented “fictional expectations” (Beckert, 2016). During the pandemic, the lockdown measure was no longer about anticipating and opening up new opportunities and horizons, but about buffering and temporarily neutralising the extraordinary nature of the singular state of shock. In lockdown, nothing is no longer about future-related expectations (planning, investments, innovations), but only about sensibly containing the social world, which has become unpredictable (survival, self-preservation). Since the extent and course of the singular crisis are unforeseeable, the future, as usual, has become uncertain and indeterminate. The time structure of a singular crisis fundamentally undermines both conservative and fictional expectations.

### 5.3 Principle of order

A third distinguishing feature pertains to the principle of social order and its temporary interruption by state authorities during the singular crisis. This interruption is limited in time, yet it marks the return of what might be called the primacy of politics, which blocks the typical market forces of the capitalist economy and all the other social mechanisms and persistent patterns of the established order. Contrary to the basic assumptions of the sociological differentiation theory that social subsystems coexist in their own logic with equal status and without a controlling centre (Luhmann, 1996), in the escalation phase of a singular crisis, a primacy of politics is to be assumed: During the pandemic, political decision-makers determined for all other fields or subsystems which measures and rules are to be applied under pandemic conditions (on “selective lockdowns” cf. Kraemer, 2021). To be more precise, the critical moment of the crisis is a primacy of the executive over the legislative and judiciary, which can be described as a “provisional state of exception” in the sense of the German philosopher and political scientist Carl Schmitt (Schmitt, 2004; see also Agamben, 2005).

In a singular crisis, the pendulum between economics and politics swings in favour of politics, regardless of whether the respective political-economic order is institutionally more market-oriented or coordinated (Hall and Soskice, 2001). In such crisis conditions, state authorities define the corridors of action until further notice. In the Covid-19 pandemic, state authorities decided whether and when to impose lockdowns, curfews, bans on the unvaccinated, or “Green Pass” requirements. They also determined which businesses and work activities are deemed systemically relevant and which are not. State authorities can also create political markets by decree, for example for face masks and test centres. And last but not least, unprecedented state aid programmes are being adopted, not only to guarantee the liquidity of specific companies affected by state-imposed lockdowns, but also to avert the collapse of whole sectors and industries. Within the European Union, these drastic interventions were financed predominantly by the European Central Bank’s bond purchase programmes, leading to a significant expansion of the ECB’s balance sheet from 2020 to 2022 (Böninghausen et al., 2022).

In this context, the primacy of politics also means that even economic dogmas that were previously considered incontrovertible or even declared sacred, such as the prohibition of indirect state financing by the ECB or a zero deficit or balanced budgetary policy (“Schwarze Null” in Germany), can be declared null and void overnight. In the sense of Carl Schmitt, a singular crisis shows that only those who can determine the state of exception and question immovable dogmas and episteme are truly sovereign in the political sense. Unintended consequences and cascade effects of these state-led interventions have become apparent, including the disruption of global supply chains that and inflationary pressures—potentially up to 50% linked to lockdown constraints (Weber and Wasner, 2023). It is important to note that this supremacy of political decisions is not driven by the usual sense of market failure observed in typical, endogenous shocks such as banking or financial-market crises in normal times. Rather, it arises from an exogenous shock that reshapes economic and political dynamics in an abrupt and far-reaching manner.

### 5.4 Social change

A fourth aspect of every singular crisis is the nature of social change. In principle, crises have the potential to lead to social and institutional changes. But crises, even singular crises, do not have to lead to transformative changes. In crises, there is no automatism and determinism. In normal times, social change tends to be gradual and incremental. In sociology, numerous social, economic, cultural and political-institutional mechanisms have been described to explain why social change tends to be slow and tough. Social persistence can delay or even block cultural or economic change. In normal times, social change can be politically postponed or even ignored for a certain period. Social change can also come to nothing if the cultural or institutional conditions for transformation are not in place. In normal times, social change can also be buffered politically and institutionally, so that the feared negative social consequences of change only become visible and noticeable with a time lag. There are many sociological reasons for non-change and social persistence. That is why social change in normal times is usually incremental rather than disruptive and transformative. In any case, political and institutional crisis intervention is always associated with non-directional, unintended social consequences. The key question is whether these are rather trivial or secondary or have a structurally formative effect.

We assume that in singular crises, the incremental social change that is typical of normal times becomes obsolete. The extent to which a singular crisis can trigger institutional change will be discussed in the final chapter. In a singular crisis, it is no longer a matter of reacting to new gradual challenges that only emerge step by step over time. Gradualism only appears to be a sensible intervention strategy as long as problems are manageable, and intermediate steps can be defined to overcome a partial problem. However, as soon as a problem constellation is perceived as overwhelming and unique and fears of complete “uncontrollability” become great, state decision-makers no longer act as moderating actors, but rather as driven and purely reactive. There is a risk that state crisis management will act as amplifiers or accelerators of an eruptive crisis. In the escalation phase of a singular crisis, it is obviously no longer so much a matter of institutional flexibility, prudence or the ability to compromise, but rather of demonstratively and performatively signalling ability to act. The collective impression is to be created from one moment to the next that there is a willingness to change track, to avert the assumed unique threat and to get the crisis under control. For example, during the coronavirus crisis, state authoritarians have temporarily suspended constitutionally guaranteed fundamental civil rights and enacted drastic non-pharmaceutical interventions to overcome the crisis.

### 5.5 Isomorphism

Fifth, singular crises differ from crises in normal times in the level or form of isomorphism. Sociological neoinstitutionalism (DiMaggio and Powell, 1983) has shown that collective actors adapt to the expectations of their social and institutional environment. Adaptation means that popular justifications (talk) and practices (action) of the social environment are imitated. However, organisations do not adapt to the expectations of their social world to increase the efficiency of the organisation. Rather, it is about not disappointing external expectations and creating legitimacy in the long term to ensure the survival of the organisation.

Sociological neoinstitutionalism does not distinguish between normal and singular crises. We argue that under the conditions of a singular crisis, isomorphism takes on a different social form and meaning than in normal times. In normal times, isomorphism is moderate or incremental, facilitated by coercive pressures, imitation of best practices, or normative influences. By contrast, the TINA principle dominates in a singular crisis. Primacy of politics means that the subordination of collective actors in all fields or subsystems is non-negotiable and therefore unconditional. In a singular crisis, isomorphism can be increased to the point of being rigorous, both institutionally and normatively. One can speak of rigorous institutional isomorphism as soon as any form of deviation or non-compliance with state guidelines threatens one’s own economic and social existence. As the coronavirus pandemic shows, in a singular crisis, institutional isomorphism can even quickly cross borders and tend to become universal, as impressively demonstrated by the rapid synchronous diffusion and astonishing homogeneity of pandemic management in the countries of the European Union well into the second year of the crisis (Sebhatu et al., 2020). In the face of a singular crisis, normative as well as institutional isomorphism can quickly take on unrelenting and strict forms. This social phenomenon of normative isomorphism became apparent during the coronavirus crisis, when individual actors were broadly delegitimised and socially marginalised as “lacking solidarity” or even as “coronavirus deniers” as soon as the appropriateness and proportionality of the measures were questioned. One example is the study by Shir-Raz et al. (2022), which examined state-orchestrated subtle practices of suppression or even censorship (‘shadow bans’, ‘strikes’) and demonisation on major private social media platforms such as Facebook, Google, YouTube and Twitter, which were by no means only directed against supporters of bizarre “conspiracy theories” (Butter and Knight, 2020), but also affected serious heterodox scientific critics of restrictive pandemic measures, such as the initiators of the Great Barrington Declaration.

### 5.6 Path dependency

A sixth important factor in understanding extranormal or singular crises involves the concept of path dependency (Pierson, 2004). Path dependency effectively extends or stretches the social phenomenon of institutional and normative isomorphism described above along the time axis. In normal times, institutional decision-making paths tend to exhibit a high degree of persistence; when they do change, the process is often incremental. Comparative political economists and social scientists studying varieties of capitalism (Hall and Soskice, 2001) or worlds of welfare (Esping-Andersen, 1990) for instance, emphasise how reforms typically unfold in a step-by-step manner. By contrast, singular crises interrupt and replace existing path dependencies, a phenomenon that can be described as a “path reset.” In such cases, a new trajectory of decision-making is introduced through political decisions, which in turn creates a fresh path dependency that unfolds along new ways.

This dynamic became evident during the COVID-19 pandemic, when measures such as border closures, school closures, lockdowns, curfews, and “Green Pass” regulations rapidly supplanted prior practices. In other words, the isomorphism of the “new normal” leads to a new path dependency. In singular crises, it is likely that state authoritarians and political decision-makers perceive a conscious deviation from the new path dependency or even a fundamental revision of the path change and a return to old decision-making paths as risky. An abrupt path change always requires justification. After a path reset, a rapid path change is rather unlikely even if, in the further course of the crisis, the general risk situation is reassessed or is considered less dramatic or catastrophic, as exemplified by the persistent adherence to strict pandemic management in Germany and Austria until well into 2022 and even in some cases until 2023.

### 5.7 Collective morality

A seventh significant dimension concerns collective morality. Here, we discuss the question of the significance of cultural factors in determining when a crisis is no longer perceived as normal but as singular. What is collective morality? We can speak of collective morality as soon as a certain perception of the world is not only shared by a relevant part of the population but is also widely circulated in the mass media as well as in public, politics and science. Such a collective morality acts as a cognitive and social filter for how the world is interpreted, which experts are consulted, which information is selected and how it is interpreted. A collective morality is hegemonic as soon as it sets the interpretive framework for what is to be regarded as “true” and what as “false,” what is to be considered “justifiable” and “dangerous” and what is to be evaluated as “risky.” It then determines what promotes the “common good.” In other words, collective morality defines the legitimate horizon of thought and action of a social group, community or even society. It is typical of such a collective morality that normative deviations are not tolerated. It has an internal integrating and external excluding effect. Max Weber (1961, p. 232) attributed such social mechanisms to the difference between “internal” and “external morality.”

We now assume that state action in a singular crisis is subordinated to one single purpose and goal—combating the singular crises. This must be culturally underpinned by a widely accepted conception of the world or episteme, which translates the extra-ordinariness of the crisis into plausible everyday narratives and emblems and meaningfully interprets the uniqueness of government crisis management. To emphasise the gravity of the situation, such a cultural model or world view must differ fundamentally from the narratives that are popular in normal times. Over the past five decades, neoliberal market narratives have typically dominated in normal times (Harvey, 2007; Deutschmann, 2019), promising individual and economic freedom, technological innovation and economic efficiency as soon as markets are deregulated, and state institutions are limited to the essentials of maintaining law and order. In the face of a singular crisis, overnight, narratives that we had assumed were almost set in stone can be replaced by other narratives that emphasise the extraordinary and incomparable nature of the crisis. The threat and danger of the situation are often highlighted, dramatic scenarios and sometimes dystopian narratives are used. The power of images is also used, as can be seen in the dramatic images in Bergamo at the beginning of the coronavirus pandemic.

In sociology, Max Weber has emphasised the pivotal significance of cultural order to explain social change like no other. Paraphrasing a famous phrase of Weber (1946, p. 280) on the social impact of “world images”—created by ideas—on social orders, the collective morality of a singular crisis can be interpreted as a cultural “switchman” of crisis management containment measures, thereby providing institutional ways along which political strategies and economic or cultural practices can be pursued in a legal and morally legitimate manner. In the case of the Corona crisis, it was the collective morality of a fear or “anxiety community” (Kraemer, 2022). From the perspective of the fear community, the pandemic has been described as a crisis that will only end well if infections are contained as rigorously as possible. In the course of the pandemic, this collective morality has developed extraordinary discursive power not only among functional elites in politics, science and the media, but also in large parts of the population. In a singular crisis, such collective morality appears self-empowering and self-legitimising. Using the example of the Corona crisis, it can be shown that the cohesive social bond of the fear community consists in the existential concern about vulnerability. Not only individuals or groups appear vulnerable, but entire societies. If we follow this world view, then all people are existentially dependent on others, ultimately on the “protecting state” (Rostalski, 2024). This collective morality appears with a self-referential gesture of superiority, which is justified by the absolute protection of the health of the community of citizens.

The collective morality of an anxiety community becomes an ultimate question of truth. This includes a strong “we-identity,” which is both inclusive within one’s own group and exclusive towards outsiders, as well as the frequent invocation of the rhetoric of TINA. Public self-commitment and widespread social mobilisation further reinforce the crisis rhetoric. Meanwhile, critics of government measures face significant pressure and are labelled as “unsolidity,” while specific groups—such as the unvaccinated—are scapegoated and stigmatised. This shift transforms the pre-crisis pluralistic debate into a binary discourse dominated by fear communities. Within this paradigm, distinctions are sharply drawn between what are deemed “facts” versus “fakes” and “truth” versus “disinformation.” Empirical evidence underscores the discriminatory dynamics inherent in such crisis narratives. Bor et al. (2023) conducted an EU-wide survey of 15,000 respondents, revealing high levels of discriminatory attitudes towards unvaccinated individuals. These attitudes, typically associated with right-wing populism in normal times and directed at migrants or ethnic minorities, were observed during the Corona crisis within liberal milieus.

### 5.8 Mode of legitimation

The mode of legitimation is an eighth distinguishing feature. Even in singular crises, the crisis management of state authorities that follow the primacy of politics is dependent on legitimisation. Compared to normal times, however, the mode of legitimisation changes in extranormal times. Now it is no longer so much a question of “input legitimation” or “output legitimation” (Scharpf, 1997). Firstly, the legitimisation of state authorities no longer depends on whether they interpret statute and law narrowly (input legitimation). And secondly, legitimacy is no longer established by the fact that the population benefits from state measures (output legitimisation). During the coronavirus pandemic, the political pandemic management decisions were rather justified in the sense of “promissory legitimacy” (Beckert, 2019) to maintain acceptance among the population and to dispel latent and manifest doubts about the meaningfulness of containment strategies. Only crisis management that promised to protect everyone seemed legitimate. The state action in a singular crisis pursues a single goal, and that goal justifies the means. In the state of exception, it suddenly becomes secondary whether the government’s crisis management still strictly adheres to the usual procedural rules and constitutional principles (input legitimisation) and whether unequal distributive effects or disadvantages for specific groups are accepted (output legitimisation).

In a singular crisis, the particular social persuasiveness of this form of legitimisation is not merely based on the promise to prevent danger and restore existential security, but on the assumption that averting danger is a matter of life or death, i.e., all or nothing. The more the risk aversion of the fear community becomes absolute, the more unlikely it is that the effectiveness of the containment measures will be subjected to an evidence test. This also explains why the foreseeable unintended collateral damage of pandemic management to the education system, particularly among children and young people, was not recognised early enough. Such mode of legitimation and such collective morality tend to become irreconcilable. From a sociological point of view, this is hardly surprising, especially since primal fears can neither be discussed nor institutionally negotiated. At this point it is useful to remember Hirschman (1994), who developed a typology of social conflicts and suggested differentiating between divisible conflicts over more or less and nondivisible either-or conflicts. Nondivisible conflicts are identity conflicts and conflicts of values that are a matter of all or nothing. Going beyond Hirschman, we argue that—as the corona crisis showed—the latent non-negotiability of fundamental value conflicts in singular crises can quickly become a manifest problem of mutual speechlessness and non-communication.

### 5.9 Spatial order

Every crisis also has a spatial dimension (cf. in normal times Löw, 2016). This is expressed in terms of where a crisis breaks out and how widespread the crisis and its consequences are. The spatial dimension of a singular crisis, we argue, differs significantly from that of a normal crisis. First of all, normal crises are localised, while singular crises are characterised by their cross-border or delimited and unbounded nature. Sociologically even more significant, however, is the difference that space in normal crises represents a neutral, simply given factor in the crisis process, while space in singular crises represents a genuinely social space and thus a space to be shaped socially and politically within the framework of crisis management. The space itself is therefore affected by the crisis and the crisis management, and spatial aspects can exacerbate or even trigger singular crises.

In cases of war, the spatial dimension of the crisis is obvious when it comes to the integrity and protection of one’s own national border or imperialist wars of conquest. The problematisation of migration movements—which in our opinion are wrongly apostrophised as a crisis—also focuses on the spatial dimension, as it involves the physical crossing of borders.

During the pandemic (Brinks and Ibert, 2020), the fact that the spatial dimension is crucial in singular crises became perfectly obvious. First of all, because the virus was transmitted from person to person, the mobility of and contact between people was identified as a problem—and one solution was seen in social distancing. In addition, publicly accessible spaces have been socially structured in a specific way during the pandemic. In normal times, the spatial organisation of society is inclusive. Inclusive means that all people have equal, free and potentially unrestricted access to public or semi-public spaces, even if access is not always unconditional, because sometimes access is only granted if a person has a certain citizenship status or can pay the entrance fee for a commercial event. However, general access bans for certain groups of people are not only unusual in normal times, but also legally inadmissible if they restrict constitutionally guaranteed civil rights. In normal times, access to public spaces is not discriminatory. As the government’s containment measures during the pandemic show—closures of or restricted access to educational institutions or public events for vaccinated and recovered people—access to public spaces, which is taken for granted in normal times, can be selectively closed or made exclusive in a singular crisis.

The dimensions and criteria analysed above provide us with an analytical tool for distinguishing between normal and extranormal or singular crises. Table 1 summarises the nine different criteria and dimensions.

**Table 1:** Criteria and dimensions of normal and extranormal/singular crises.

	Normal crises	Extranormal/singular crises
Involvement and impact	Limited	Unbounded
Temporality	Flowing times	Whiteout moment
Principle of order	Primacy of economy	Primacy of politics
Social change	Incremental	Eruptive
Isomorphism	Medium	TINA
Path dependency	Path persistence	Path reset
Collective morality	Liberal market narrative	Anxiety community
Mode of legitimation	Input- and output-legitimation	Legitimation through promises
Spatial order	Neutral spatiality	Social and designable spatiality

## 6 Conclusion: singular crises and institutional change

In the previous sections, we looked at the concept of crisis and the distinction between normal and extranormal or singular crises. Using the example of the coronavirus pandemic, we have shown that singular crises are indeed unique, but at the same time have characteristic social patterns that distinguish this type of crisis from normal ones. While a normal crisis occurs cyclically and usually leads to incremental change, a singular crisis is characterised by eruptive ruptures which challenge the traditional social order, both institutionally and narratively. Unlike a normal crisis, a singular crisis is marked by exogenous shocks like wars, natural disasters, or pandemics. By using the Covid-19-crisis as an empirical slide, we identified nine dimensions and criteria—namely involvement and impact, temporality, principle of order, social change, isomorphism, path dependency, collective morality, mode of legitimation and spatial order—that can be used to differentiate between singular and normal crises.

At the peak of a singular crisis like the corona crisis, it almost seems as if a central structural principle of modern (capitalist) societies, functional differentiation, is being temporarily called into question. One can even speak of a partial or relative de-differentiation of politics, economics, law, science and mass media, whereby at least three phases can be distinguished. In the first escalation phase, the population rallies behind the state authorities and decision-makers, who declare a cross-field or cross-subsystem state of emergency. In the moment of shock of the crisis, the external threat leads to internal conformity (“rally ‘round the flag”). As soon as the first shock moment has passed, an internal opposition to the declared state of exception is formed in the second phase of a singular crisis, which in turn is publicly delegitimised and stigmatised by state and state-affiliated actors in politics and the mass media (“corona denier”). In a state of exception, state authorities function even more as legitimate representatives of monopolised “symbolic power” (Bourdieu, 2015). In the third phase, the finally delegitimised opposition radicalises itself by looking for simple thinking to “explain” the crisis management of state authorities in everyday life (“conspiracy myths”). Ultimately, the politically declared provisional state of exception at least partially affects the functionally differentiated normality mode of politics (government vs. opposition), public (opinion vs. counter-opinion), science (evidence vs. counter-evidence), economy (material vs. formal rationality) and law (balancing of rival legal rights).

Finally, we want to draw some theoretical conclusions on a sociology of singular crises and problematise whether recurring social patterns can possibly be identified in post-singular crisis phases. The special focus here is on the unresolved question of whether singular crises are collectively forgotten as quickly as they occurred or whether they trigger or accelerate far-reaching institutional change.

What can sociology actually do in relation to singular crises and what is the contribution and task of sociology? With regard to singular crises, the task of sociology is twofold: On the one hand, it is about developing a general theory of singular crises—in other words, providing the analytical-conceptual tools for empirical analysis of singular crises, to identify recurring social patterns in singular crises and to distil typological criteria with which singular crises can be distinguished from normal crises.

On the other hand, it is about concrete empirical analysis in the shock moment of a singular crisis. Here, the aim is to explain the causes, the consequences and the possible paths of development after the crisis. A sociology of singular crises should identify, diagnose, and criticise the structures, mechanisms, actions, and conditions that generate the singular crisis in the first place. In addition, a sociology of singular crises must critically analyse the crisis management and reflect on the dominant narratives during the crisis. Using the example of the coronavirus pandemic, we argue that sociology, instead of falling into the mode of crisis rhetoric of simple words, should remember the methodological and methodical core competences of the discipline to critically analyse the crisis and the crisis management of the state (Kraemer, 2023).

We have argued that the corona crisis is particularly suitable for the sociological study of social patterns of a singular crisis. The next step would be to examine whether other crises can be identified that exhibit similar social patterns to the singular corona crisis. A further step would be to analyse historical crises along a continuum between 0 and 1, where 0 stands for normal crises and 1 for singular crises. We suspect that the July Crisis 1914, the Paris May 1968 or the German Autumn 1977 would be well suited as case studies to further develop our considerations into a sociological theory of singular crises. In this article, however, we cannot provide a comparative historical-sociological analysis of different crises to test the fruitfulness of the proposed typology of normal vs. singular crises. Instead, we would like to conclude with some brief general remarks on the post-crisis of the singular corona crisis and problematise the extent to which a singular crisis can trigger institutional change.

Nobody can withstand a state of exception over a long period of time. As soon as daily crisis reporting in the mass media fades into the background (cf. Robertson and Doshi, 2021), the crisis narrative also becomes obsolete. Depending on the constitution of the institutional order, the path-dependent consequences of crisis management and the receptiveness of the political culture to institutional learnability, the exit from the state of exception will be faster or slower. In addition, the institutional learning curve is influenced by social-psychological phenomena such as sunk cost effects (Janssen et al., 2003), based on abilence paradox (Harvey, 1974), escalating commitment (Staw, 1976), threat-rigidity effects (Staw et al., 1981) and groupthink (Esser, 1998) or even strategic ignorance (McGoey, 2012), can even be permanently blocked by social patterns of collective repression and forgetting as well as by a lack of retrospective political reappraisal of the crisis and the crisis measures politically (Schippers et al., 2024). In any case, the time of narrative and institutional re-normalisation is heralded as soon as a general collective crisis exhaustion spreads. It is an unanswered empirical research question whether a singular crisis heralds lasting institutional change or whether this does not materialise. Theoretically, different trajectories are conceivable in the post-phase of a singular crisis, depending on various social, structural, economic, political-institutional and cultural factors of the pre-crisis period, which we cannot discuss in more detail in this paper.

In conclusion, we want to distinguish between three possible scenarios: The first scenario would be an unrestricted return to the old normality. In this case, despite the manifest economic, social and political upheavals, the singular crisis remains almost without consequences for further long-term development. In this scenario, the wounds left by the singular crisis may not heal over time, but sooner or later they will be institutionally and socially forgotten.

The second scenario is the opposite of the first. In this case, a return to the old normality is blocked even in the post-phase. The new path-dependent normality of the singular crisis becomes a permanent institutional state that will not go away even if the actual singular crisis is already far behind. In the second scenario, the singular crisis acts as an impetus for transformation, as a driver of change and, according to the original meaning of crisis in Greek antiquity, as a “turning point” (Abbott, 2001; Sewell, 1996) which sets a new direction for future political, institutional, economic, and social development that was hardly conceivable before the outbreak of the singular crisis.

The third scenario is located between the first and second scenario. In this scenario, the singular crisis acts as a reinforcement or acceleration of longer-term trends in politics, culture, and society. These were already foreshadowed in the pre-crisis, but without unfolding their impact in full breadth and depth. It is only during the singular crisis that they fully come to the surface. They not only characterise acute crisis management but also continue to shape future post-crisis action in politics and state order. Ultimately, it is an open empirical research question as to what remains of a singular crisis.

## Footnotes

^1^For the definition and history of the concept and meaning of crisis, see Steg (2020, 2023). The following section refers primarily to these sources. See also Koselleck (1988, 2006); Habermas (1992); and Roitman (2014).
